# Molecular species delimitation of a symbiotic fig-pollinating wasp species complex reveals extreme deviation from reciprocal partner specificity

**DOI:** 10.1186/s12862-014-0189-9

**Published:** 2014-09-18

**Authors:** Clive T Darwell, Sarah al-Beidh, James M Cook

**Affiliations:** School of Biological Sciences, University of Reading, Reading, RG6 6AS UK; Department of Biology, Syracuse University, 107 College Place, Syracuse, NY 13244 USA; Royal Horticultural Society, Wisley Garden, Woking, Surrey, GU23 6QB UK; Hawkesbury Institute for the Environment, University of Western Sydney, Locked Bag 1797, Penrith South DC, NSW 1797 Australia

**Keywords:** Species delimitation, Agaonidae, *Ficus*, Pollinator, Wasp

## Abstract

**Background:**

Symbiotic relationships have contributed to major evolutionary innovations, the maintenance of fundamental ecosystem functions, and the generation and maintenance of biodiversity. However, the exact nature of host/symbiont associations, which has important consequences for their dynamics, is often poorly known due to limited understanding of symbiont taxonomy and species diversity. Among classical symbioses, figs and their pollinating wasps constitute a highly diverse keystone resource in tropical forest and savannah environments. Historically, they were considered to exemplify extreme reciprocal partner specificity (one-to-one host-symbiont species relationships), but recent work has revealed several more complex cases. However, there is a striking lack of studies with the specific aims of assessing symbiont diversity and how this varies across the geographic range of the host.

**Results:**

Here, we use molecular methods to investigate cryptic diversity in the pollinating wasps of a widespread Australian fig species. Standard barcoding genes and methods were not conclusive, but incorporation of phylogenetic analyses and a recently developed nuclear barcoding gene (ITS2), gave strong support for five pollinator species. Each pollinator species was most common in a different geographic region, emphasising the importance of wide geographic sampling to uncover diversity, and the scope for divergence in coevolutionary trajectories across the host plant range. In addition, most regions had multiple coexisting pollinators, raising the question of how they coexist in apparently similar or identical resource niches.

**Conclusion:**

Our study offers a striking example of extreme deviation from reciprocal partner specificity over the full geographical range of a fig-wasp system. It also suggests that superficially identical species may be able to co-exist in a mutualistic setting albeit at different frequencies in relation to their fig host’s range. We show that comprehensive sampling and molecular taxonomic techniques may be required to uncover the true structure of cryptic biodiversity underpinning intimate ecological interactions.

**Electronic supplementary material:**

The online version of this article (doi:10.1186/s12862-014-0189-9) contains supplementary material, which is available to authorized users.

## Background

Symbiosis between disparate organisms has been responsible for key innovations in evolutionary history, such as the origin of plants [1]. It is also pivotal in several vital ecosystem functions, such as nitrogen fixation [2] and pollination e.g. [3], and has been implicated as a major driver in the generation of biodiversity [4,5]. The intimate nature and high partner specificity of many symbioses establishes conditions for strong interactions between the species involved, in terms of both population dynamics and coevolution [6,7]. A basic but important question is “how many species are involved in a host/symbiont interaction?”, because species interaction strength, a major determinant of ecological and evolutionary dynamics, will differ between a system with one host and one symbiont species and one with multiple host or symbiont species [8]. In addition, when multiple symbionts compete to utilise host resources, this may select for more selfish or virulent behaviour.

The obligate mutualism between fig trees and their pollinating wasps is a classic and much-studied example of symbiosis [9-11]. Fig trees are important components of rainforest and savannah ecosystems and provide food for many vertebrate and invertebrate animals [12]. Figs can only be pollinated by host-specific wasp species, which, in turn, can only reproduce by laying their eggs in fig flowers, upon which their developing larvae feed. This association was long considered to be a textbook example of extreme reciprocal partner specificity, with each fig species having a unique pollinator species [9]. However, this picture has been eroded by several reports of a single fig species hosting multiple pollinator species and a few reports of two fig species sharing pollinator species [13-21].

It is now clear that the paradigm of one-to-one reciprocal partner specificity is no longer tenable [9,22], but true patterns of partner species associations remain to be revealed. Recent reviews have posited that perhaps a third or half of the >750 fig species worldwide may have multiple pollinator species [9,23]. However, these estimates are best regarded as informed guesses for two main reasons. First, most of the evidence for multiple pollinators is the by-product of studies conducted for other reasons, such as exploring wasp reproductive behaviour [15], or phylogeographic histories [18]. Thus, these lines of evidence do not come from targeted studies aiming to document the numbers of species involved in the plant/pollinator symbiosis. In particular, there is a striking lack of studies involving wide sampling of insects from across host plant geographic ranges [19,24].

Second, there is a “taxonomy gap” that hinders assessment of species associations [25-27]. There are only about 150 described agaonid species, but the true number seems likely to exceed 1000 [28]. This means that investigation of the pollinators of many fig species begins with only a genus level identification and with no described wasp species already linked to the fig species in question. Moreover, even when a described pollinator is known, genetic studies often reveal further pollinator species that are either morphologically cryptic within the one named entity, morphologically distinguishable but not previously sampled, or previously sampled but unrecognised within mixed species collections, e.g. [29].

Molecular data have already contributed substantially to rejection of the old 1:1 paradigm of fig/pollinator specificity and should now play a key role, in tandem with morphological analysis, in establishing true patterns of pollinator diversity and variation in plant/pollinator interactions at local and regional scales [19]. Wide geographic sampling is crucial [30], because: a) some species may not occur in all parts of the host range, and b) if intraspecific genetic variation is underestimated by sampling few sites, it may be harder to identify the molecular ‘barcoding gap’ (or appropriate clades in phylogeny-based methods) between species [31,32].

Another important issue is the choice of markers for molecular taxonomy. The animal barcoding approach developed by CBOL uses a standard section of the mitochondrial COI gene [33,34]. This has proved valuable in some studies of fig wasps, but in a recent phylogenetic study of 200 species from 19 genera [35], the success rate for PCR and direct sequencing was much higher with cytochrome b and these two linked mitochondrial markers tend to reveal very similar patterns [36]. Both markers can vary substantially within fig wasp (and other) species, which may hinder correct species delimitation, in particular via over-splitting [37,38]. Consequently, it is desirable to also use a nuclear marker and to seek congruent species delimitation between the two genomes [39]. With nuclear markers, the challenge is to find one that both amplifies reliably across species and shows sufficient variation for discrimination between closely related species [40]. None of the currently used nuclear markers achieve the widespread utility of favoured mtDNA markers like COI and cytb. However, an internal transcribed spacer region (ITS2) of rDNA has recently been proposed as a useful nuclear barcoding marker for animals [41-44].

A further issue with molecular data is the choice of species delimitation method. Barcoding-type approaches use genetic distance data directly to identify a barcoding gap between the pairwise genetic distances found within and between species. These approaches often work well in practice, but threshold genetic distances vary across taxa, and well-known biological phenomena, such as introgression and selective sweeps due to *Wolbachia* bacteria, can confound the expected patterns. Meanwhile, phylogeny-based methods aim to identify clades that are evolutionary significant units (ESUs) and invoke the phylogenetic species concept [45]. These have a justifiable conceptual basis, but are yet to be as widely used and are experiencing a period of relatively rapid methods development.

In this study, we investigate the diversity of pollinator wasps associated with a single widespread fig species, *Ficus rubiginosa.* This plant is endemic to Australia and occurs widely in diverse habitats, including eucalypt scrub and rainforest, in a roughly 2500 km coastal belt that stretches from tropical northern Queensland to temperate southern New South Wales [46]. It belongs to the *Ficus* section Malvanthera, which is pollinated by wasp species in the genus *Pleistodontes*. Its only known pollinator species is *P. imperialis,* which was originally described by Saunders (1882). Following new wasp collections from several malvantheran fig species, a taxonomic revision of *Pleistodontes* was carried out [47]. This led to the description of seven new *Pleistodontes* species overall, but no change to the conclusion that *F. rubiginosa* was associated with a single pollinator species (*P. imperialis*)*.*

Subsequent genetic work by Haine et al. [17] involved sampling *P. imperialis* from several regions with the aim of investigating the phylogeography of the species. Instead, cytb data revealed four deep clades, suggesting the presence of cryptic species. Nuclear sequence data were obtained from the D2 region of 28S rDNA, but this showed almost no variation between individuals in the samples. A second nuclear region, wingless, was also studied and again proved almost invariant. The authors concluded that, despite lack of resolution from the nuclear markers, the data supported the existence of four species within the ‘*P. imperialis’* complex*.* Re-examination of insect specimens from each clade by an expert taxonomist (J-Y Rasplus, INRA) revealed that one putative species (clade two in [17]) could be distinguished by morphology (colour) alone, and another (clade 1) by relative head length, demonstrating the value of integrating molecular and morphological information and iterative assessment of species boundaries e.g. [48]. However, two further putative species (clades 3 and 4) remain morphologically cryptic. *F. rubiginosa* therefore has one of the highest diversities of pollinator species known for any fig species [18,19,49]. However, as in other studies, this has been revealed as a by-product of sampling wasps for other purposes. Here, we make a targeted study with the aim of using extensive sampling to:

1. Compare the 'performance of mitochondrial (cytb and COI) and nuclear (ITS2) molecular markers, and different species delimitation methods (distance and phylogeny-based) for identifying species.
2. Uncover the full diversity of pollinator species associated with *F. rubiginosa* and the impact of sampling effort on their detection*.*
3. Explore geographic turnover (beta diversity) of pollinator species across the host plant’s wide natural latitudinal range.

## Results

**Figure 1:**
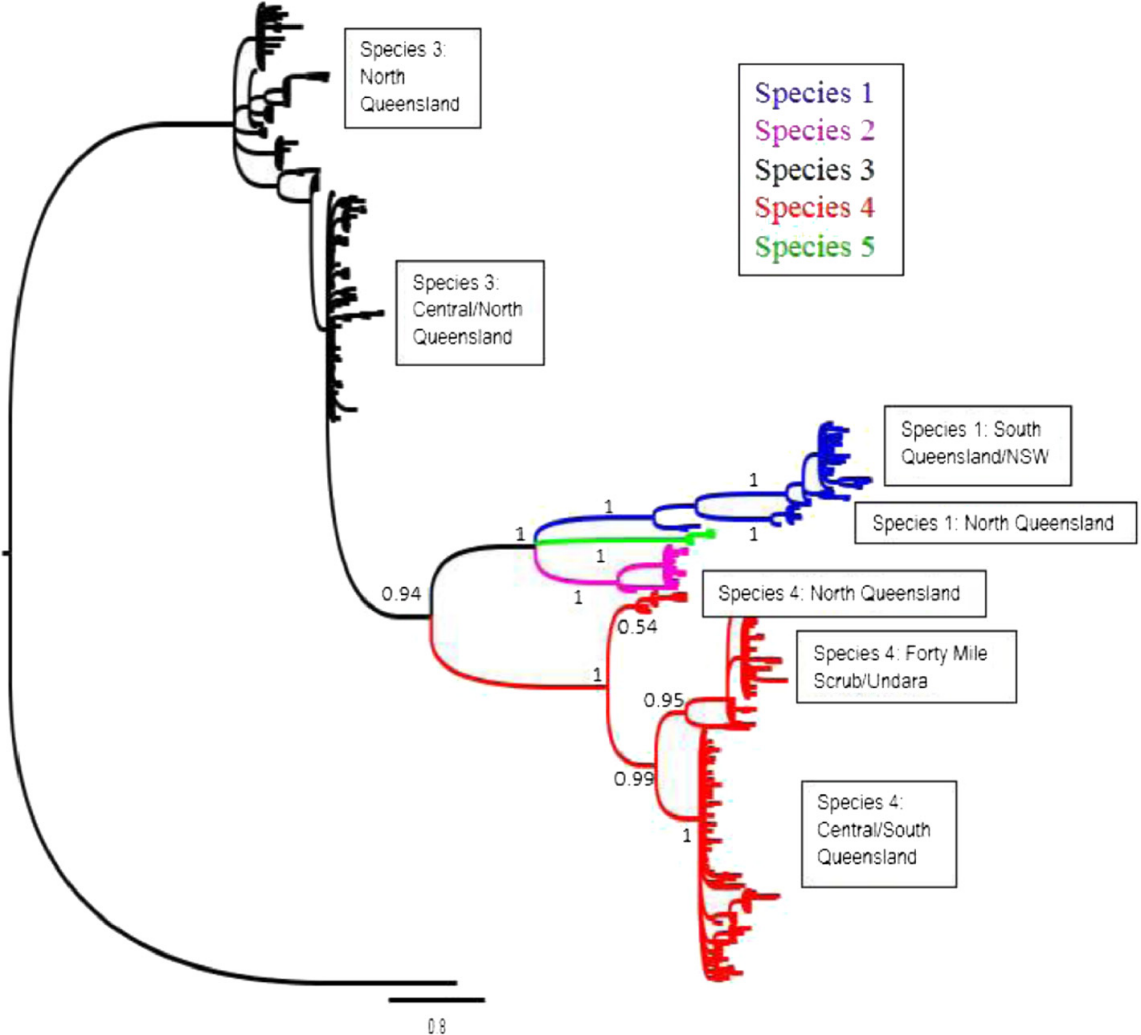
**Schematic diagram of Bayesian cytb phylogeny of 415** ***P. imperialis*** **individuals.** Colours indicate five putative species though sub-clades indicate up to 11 ESUs. Posterior node probabilities are indicated for the five species (see Supplementary Information for annotated phylogeny).

Bayesian phylogenetic analysis of 415 *Pleistodontes imperialis* cytb sequences identified five species when following the circumscription of Haine et al. [17] (Figure 1 and Additional file 1: Figure S1). These are the four hypothesised species found by Haine et al. [17] and one new species. Support values (p = 1) are high for all five of these main clades. However, a case can be made for up to 11 significant clades (Figure 1), because there are distinct sub-clades within the main clades. Species 1, 2 and 3 each exhibit two sub-clades and species 4 exhibits three. The different geographical distributions of these sub-clades (with northern and southern sub-clades in three of the species – see below) further suggest a hypothesis of additional taxa, above and beyond those identified by Haine et al. [17], as independently evolving, non-introgressing lineages.

**Figure 2:**
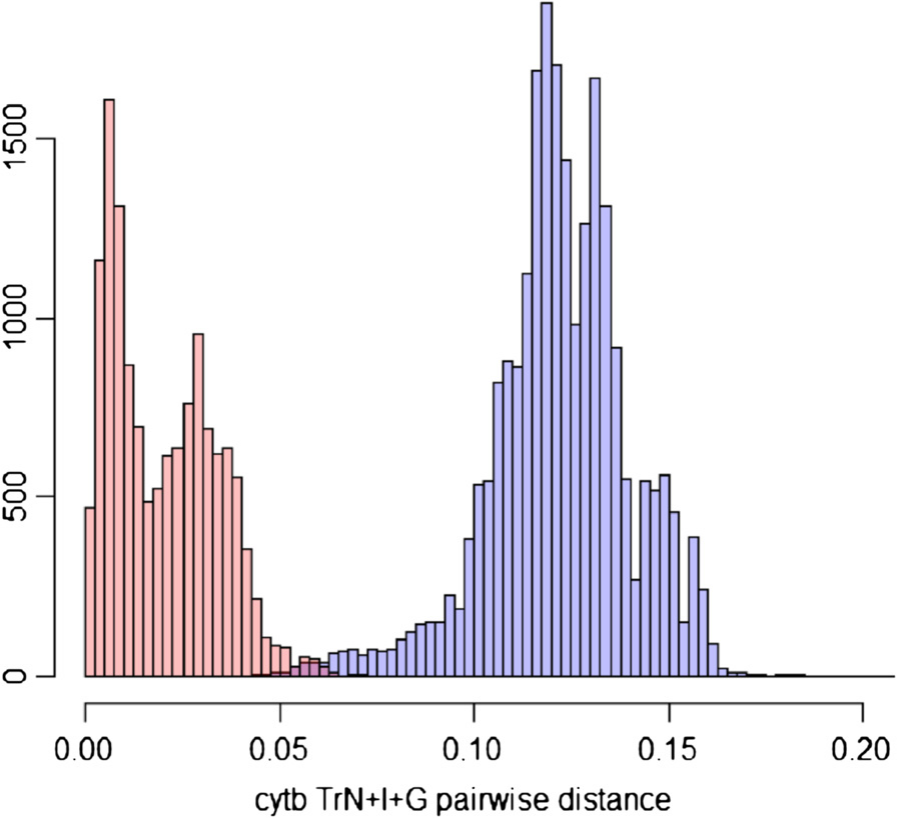
**Modelled TrN + I + G pairwise distance distribution for 415** ***P. imperialis*** **cytb sequences.** No barcode gap is evident. Intraspecific distances range between 0–7.2%; interspecific distances between 4.3-18.3%.

**Figure 3:**
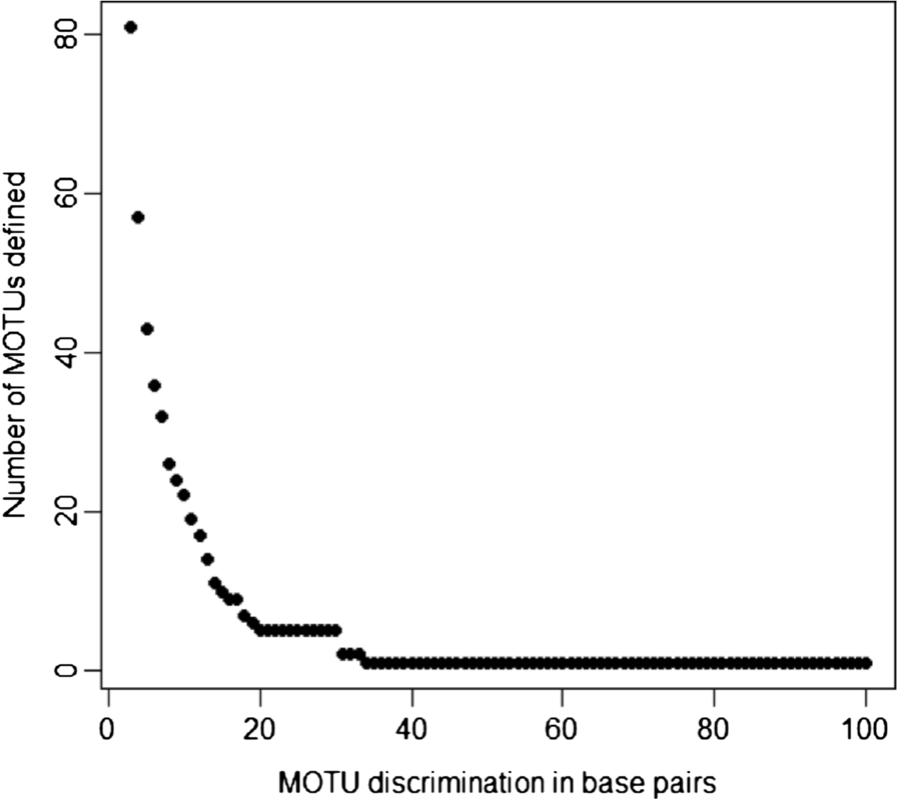
**Output from jMOTU suggests five** ***P. imperialis*** **MOTUs according to percentage sequence discrimination threshold employed for cytb sequences.** Plateau between 17–30 bp indicates barcoding gap.

Under a five pollinator species hypothesis, visual investigation of pairwise distances (Figure 2) does not reveal a clear barcode gap in cytb data as intraspecific divergences (0–7.2%) overlap slightly with interspecific divergences (4.3-18.3%). However, analysis of cytb data using jMOTU does favour five species (Figure 3) with a barcode gap between 17–30 base pairs discrimination. GMYC analyses on cytb data also indicated the existence of five species. The GMYC model was preferred over the null model of uniform branching rates (GMYC logL = 304.34, null model logL = 297.69, p < 0.01). All of the 330 analysed individuals are placed in the same species by both the jMOTU barcoding and GMYC approaches using the cytb gene.

Bayesian analysis on a subset (n = 44) of individuals for the 416 bp COI region suggests six major clades (Additional file 2: Figure S2), comprising the five major cytb clades but with species 1 split into northern and southern clades. However, support values for clades 2–5 are all 1.00 whilst the two species 1 sub-clades have support values of 0.53 and 0.80 indicating poor support for a six species hypothesis. Moreover, jMOTU analysis of COI indicates five species (Additional file 3: Figure S3). Visual investigation of pairwise distances (Additional file 4: Figure S4) also does not reveal a clear barcode gap in COI data, with intraspecific divergences of 0–5.6% overlapping slightly with interspecific divergences (5.0-13.1%). Sub-clade congruence is absolute for individuals sequenced for both cytb and COI.

**Figure 4:**
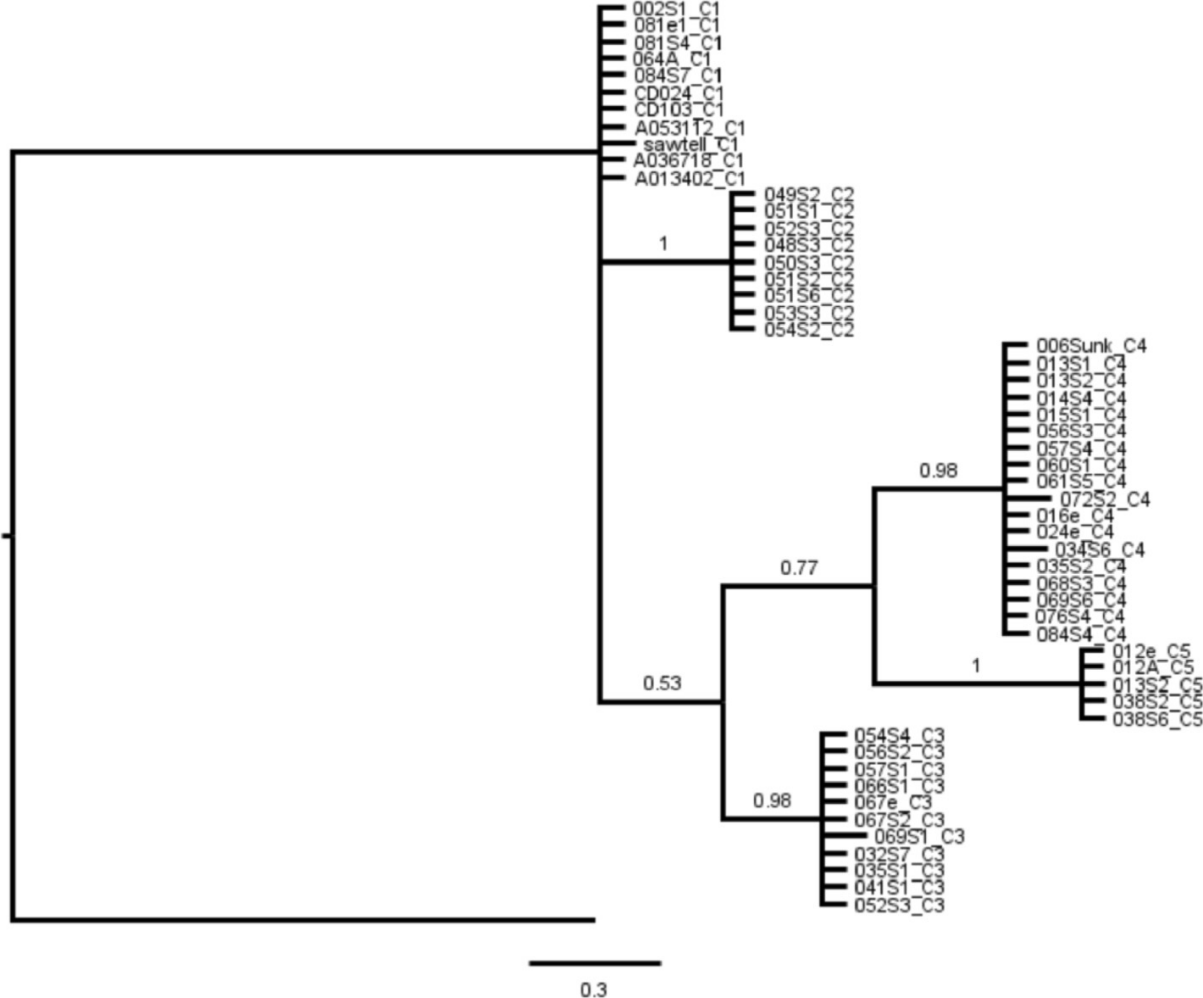
**Consensus Bayesian topology from ITS2 data for 54** ***P. imperialis*** **individuals.** Posterior probabilities are indicated. Tip suffices denote major cytb clade (i.e. species) assigned to each individual by cytb analyses (e.g. C1 = cytb species 1).

Bayesian phylogenetic analyses of ITS2 data also support a hypothesis of five species (Figure 4). However, in contrast to the cytb analysis, there is a marked lack of sub-clade structure. Indeed, most individuals within each of the five species have identical sequences, while there are substantial differences between species, making species placement unequivocal using this marker. Support values are high for all hypothesised species nodes. Again, all species placements are congruent with those determined by cytb analyses.

**Figure 5:**
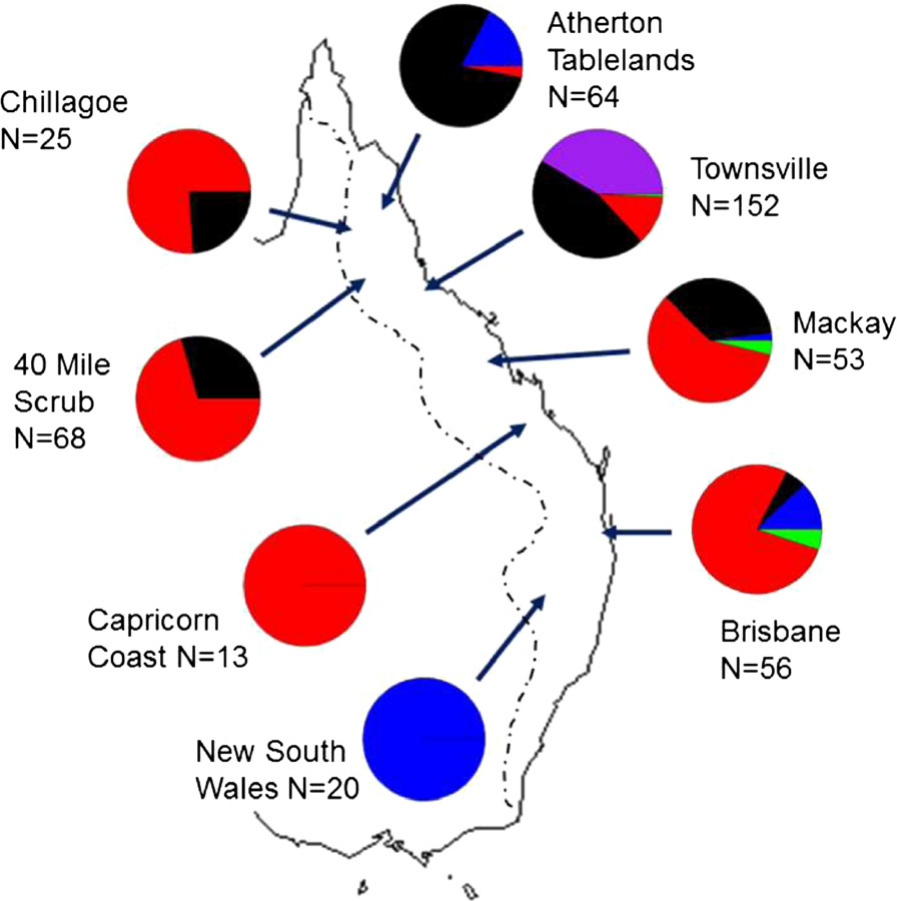
**Distribution and frequencies of five** ***Pleistodontes imperialis*** **species pollinating the fig,** ***Ficus rubiginosa*** **, in eastern Australia.** N denotes sample size of geographic region. Townsville region includes 43 morphologically identified yellow pollinators. The dotted line indicates the geographic range of *F. rubiginosa* modified from [46].

Figure 5 shows the frequencies of pollinators belonging to each of the five *P. imperialis* species at sites across eastern Australia (see Additional file 5: Table S1 for values). Chi-squared tests show species to be unevenly distributed across populations (*χ*^2^ = 558.6048, df = 28, p < 2.2×10^−16^). This pattern is also evident when testing the four most common species individually (species 1: *χ*^2^ = 78.1282, df = 7, p = 3.315×10^−14^; species 2: *χ*^2^ = 441, df = 7, p < 2.2×10^−16^; species 3: *χ*^2^ = 203.3034, df = 7, p < 2.2x10^−16^; species 4: *χ*^2^ = 121.8537, df = 7, p < 2.2x10^−16^). Most sites have more than one (and up to four) pollinator species present, so there is no absolute geographical replacement of species. Nevertheless, three species (1, 3 and 4) each dominate in particular regions, whilst species 2 is co-dominant with species 3 in Townsville.

## Discussion

Symbioses have been implicated in the innovation of key evolutionary leaps, the development of a number of fundamental ecosystem functions, and as major drivers in the generation of biodiversity. However, the exact nature of the ecological relationships between symbiotic partners is often unknown due to the lack of targeted investigations, taxonomic impediments and insufficient sampling. Among classic examples of symbioses, the fig-wasp system provides a crucial ecological resource across tropical and savannah environments. Here we provide the first comprehensive targeted investigation to assess pollinator diversity across the geographic range of a common and widespread fig species. Our study supports the existence of at least five wasp species pollinating *Ficus rubiginosa*. This *P. imperialis* complex involves more pollinator species than any other fig species studied to date [18], but see [21,49] and our study adds to the growing body of evidence that the diversity of fig wasps, and insects in general, has been greatly underestimated [9,23,50].

Our most comprehensive sampling used mitochondrial cytb and delineated up to 11 ESUs that might each constitute a species (Figure 1). This cytb diversity is far greater than previously identified and is most likely explained by a big increase in sampling effort relative to that of Haine et al. [17], whose analyses highlighted four largely unstructured clades. Most striking among this new diversity are the six individuals ascribed to species 5 that are distinguished from other species by extended branch lengths in the phylogeny. These most likely represent a rare fifth pollinator species not sampled previously. Moreover, we found strong sub-clade structure within some of the putative species and this generally corresponds to largely or wholly Northern versus Southern populations of the more widespread species. Such geographic isolation is suggestive of restricted gene flow and could therefore indicate distinct species, but further results argue against this.

Investigation of modelled pairwise genetic distances for cytb data suggests that species delimitation is not straightforward among these wasps due to the lack of a barcoding gap. However, jMOTU analyses of cytb data clearly supported the existence of five cryptic species and this algorithm identified a barcoding gap between 17–30 base pairs. Additionally, GMYC analyses on cytb data give strong statistical support for the existence of five *P. imperialis* pollinators. This method adds valuable weight to the five species hypothesis as it statistically tests the crossover point between intra- and interspecific branch lengths derived from genetic markers.

In order to compare cytb phylogeny with the standard cox1 barcoding gene, a shortened COI fragment of only 416 bp cf 658 bp; [33] was employed on a subset of 44 individuals. The COI topology is very similar to that for cytb but splits species 1 into two. However, the very low support values (p = 0.53 and p = 0.80) offer no strong evidence that either of the species 1 sub-clades are more closely related to species 2 for this marker. Moreover, jMOTU analyses support five species for this marker in the same way as for cytb. The nuclear ITS2 phylogeny is similar to that for cytb in revealing the same major ESUs. However, sub-clade structure is absent for this marker and there is essentially zero variation within any of the five species, allowing straightforward delimitation of taxa. Interestingly, this means that ITS2 behaves far more like a barcode in the wider general sense of this word than mtDNA markers like COI or cytb, which typically show considerable within species variation even when there is a clear barcoding gap. This disparity in resolution between mtDNA and nDNA is most likely explained by different population genetic properties of these markers [51], and by multigene concerted evolution of the repeated rDNA cluster.

Our sampling reveals that each of the five species has a different geographic range (Figure 5). Whilst there is no absolute geographical replacement of species, four species either dominate or are codominant in particular regions. Species 3 is dominant in northern Queensland, while species 4 appears dominant in central and southern Queensland and in the inland region around Forty Mile Scrub. The yellow species 2 is found only in the Townsville region, where it is common and co-occurs with species 3 at similar frequencies. Finally, species 1 occupies widely disjunct regions in the far north of the host plant range and in New South Wales, where it is the only pollinator species recorded. Moreover, species 1 appears excluded from most of the intermediate regions as no individuals were recorded amongst 220 wasps from the Townsville and Forty Mile Scrub populations. Only species 5 appears to have no geographical stronghold, with only six individuals sampled overall. However, this may be an artifact of the low sample size for this rare species.

From the perspective of understanding the ecological dynamics of symbiosis this study clearly rejects the simple model of reciprocally partner-specific fig and pollinator species. Over most of the host plant range, it is associated with more than one pollinator species, but the frequency of the different species varies considerably between regions. Given the wide latitudinal range of the host plant, from the wet tropics to the temperate zone*,* it is possible that pollinator diversity is related to local adaptation to different climates. Whatever the causes of variation in pollinator identity and diversity between regions, the variation itself has important implications for insect/plant coevolution [52]. First, the association appears to be a 1:1 match of a fig and pollinator species in the southern part of the range in NSW, but to typically involve two or three (but not always the same) co-occurring pollinator species at sites further North in Queensland. This could lead to different coevolutionary trajectories in different parts of the range, e.g. because multiple symbiont species may increase host/symbiont conflict as a result of antagonistic competition between the symbiont species [8].

Another set of questions arising from our improved understanding of pollinator species diversity and distributions surrounds the local coexistence of multiple pollinator species utilizing the same host resources. Ecological theory suggests that this is difficult to achieve without some degree of niche separation, although an intriguing possibility with fig wasps is that it might occur through density-dependent sex ratio (and therefore population growth rate) variation [53]. Different sites have different sets of pollinators (Figure 5), creating the possibility to compare different coexistence patterns in different but overlapping sets of species.

There has been only limited investigation of the comparative ecology of multiple sympatric pollinator species associated with a given fig species [13,15-18,54] and most such studies have not revealed clear differences. Two exceptions are cases where one of the pollinators is a “cheat” that does not actively pollinate its host plant [55,56], and cases where one species is a diurnal, and the other a nocturnal, disperser e.g. [57]. The latter case may be relevant in the *P. imperialis* complex because species two is yellow rather than dark brown or black, and previous studies in Africa and Asia have shown that pale coloured wasps tend to be nocturnal dispersers, while dark ones tend to be diurnal e.g. [57]. This is one potential axis for niche differentiation, but four of the five *P. imperials* complex species are brown/black wasps so are unlikely to be differentiated in this way. Further research into host use by the *P. imperialis* species complex could focus on genetic and morphological variation within *F. rubiginosa*.

Another key message from our study is the importance of a comprehensive sampling regime. Haine et al. [17] sampled 71 *P. imperialis* from across *F. rubiginosa*’s range and hypothesised the existence of four cryptic species. Our sampling covered much of the same geographic region in a more thorough manner, as well as some additional locations. Despite sampling 415 wasps, we only captured six individuals of the newly identified species 5 from three coastal sites up to 1000 km apart in central and southern Queensland. It remains possible that further ‘*P. imperialis*’ species are yet to be sampled. The sampling effort from NSW is much less comprehensive than that of Queensland, but this so far appears justified as only a single species has been found from 20 specimens from several sites.

## Conclusions

Our study offers comprehensive evidence that mutualistic *P. imperialis* wasps pollinating the fig species *F. rubiginosa* have diversified into five distinct (including some cryptic) species across their entire host plant range along the east coast of Australia. Given the absence of diagnostic morphological and ecological differences in this sister-species complex that may offer corroborating evidence for the assessment of species status in an integrative taxonomic framework, we have shown the utility of the internal transcribed spacer region (ITS2) of rDNA as a diagnostic tool for species delimitation among problematic taxa. Furthermore, we have shown that four out of five of these identified taxa show strong patterns of regional geographic dominance that suggests an adaptive origin to localised ecological conditions and offers a likely mechanism in mediating their co-existence performing the same ecological role in the same fig species. Moreover, where we identify a pattern of geographic co-dominance there is reason to believe that variation in pigmentation may indicate niche differentiation in diurnal/nocturnal dispersal patterns. In addition to this interspecific geographic structuring, our comprehensive sampling also suggests geographic structure at the intraspecific level for three of the species. Thus, our results show that one-to-one specificity may often break down in symbioses and offer an explanation as to under what circumstances it may do so. Additionally, the increasing level of pollinator diversity at lower latitudes implies that coevolutionary trajectories between host and symbiont will vary across the range. In summary, our understanding of the structuring of biodiversity in a symbiotic context is likely to be simplistic and will require targeted studies using molecular taxonomic techniques and comprehensive sampling schemes if we are to better understand the coevolutionary dynamics underpinning intimate ecological interactions.

## Methods

### Sample collection

Most sampling was conducted in Queensland from 2007–2009 along the eastern seaboard and immediate hinterland between Brisbane (26° 46S, 153°02E) in the south and Dimbulah (17° 01S, 145°19E) in the north. Sporadic sampling was also undertaken in New South Wales (NSW), and also from some planted trees outside their natural range in Victoria and South Australia between 2000 and 2010. Near-ripe figs were collected from trees and placed into hatching jars with meshed lids that allowed air flow, prevented overheating, and prevented wasp escape. After 48 h each fig and all its emerged wasps were placed into 70% ethanol. Alternatively, figs were placed directly into alcohol and wasps were dissected out at a later date. Since most pollinator wasps developing in a given fig are siblings, we used only one wasp per syconium for DNA extraction to maximise the independence of samples. Sampling intensity was greater in Queensland than in New South Wales, but evidence for much lower diversity in New South Wales both before [17] and after this study (T. Sutton, *pers. comm.*) justifies the lower sampling effort in NSW. Additional file 6: Table S2 gives location, markers sequenced and GenBank accession numbers for all 415 wasps used in this study.

### Molecular methods

A Chelex method was used for DNA extraction [58] and two mitochondrial (cytb and COI) and one nuclear (ITS2) marker were amplified. NB the COI marker is a region of cox1 shorter than the standard barcoding region, where alternate primers have been designed to avoid nuclear pseudo-genes within certain taxa: [59]. Our widest genetic sampling of individuals focussed on cytochrome b (cytb) using mtDNA primers CB1 and CB2 [60]. A 396 bp fragment of cytb was amplified for 415 wasps in order to assign individuals to the putative species identified by Haine et al. [17]. Cytb was chosen as our primary marker in order to integrate our study with the earlier one by Haine et al. [17]. We also analysed a subset of individuals using the COI region to facilitate comparison between mtDNA genes and connectivity with other datasets.

Subsets of 3–15 individuals per cytb clade were sequenced for ITS2 (n = 54) [61] and a 416 bp fragment of COI (n = 44) (primers CI-J-1751 and CI-N-2191; see [62]) in order to clarify and confirm species delimitation. Cytb was amplified using a Techne Touchgene gradient machine with 3 min at 94°C, 30 cycles of 15 s at 95°C, 20 s at 45°C, 30 s at 72°C, and a final elongation step of 10 min at 72°C. Amplification of COI consisted of 5 min at 94°C, 30 cycles of 30 s at 94°C, 45 s at 50°C, 60 s at 72°C then 10 min at 72°C. Amplification of ITS2 used 5 min at 94°C, 35 cycles of 30 s at 94°C, 40 s at 55°C, 40 s at 72°C then 10 min at 72°C. Subsequent purification and sequencing reactions were conducted by Macrogen Inc. Purification was performed using ethanol precipitation and sequencing by BigDyeTM terminator cycling conditions and a 3730xl DNA analyser. All sample sequences have been deposited in GenBank: cytb [GenBank: KM249475-KM249848], COI [GenBank: KM249375 - KM249419], ITS2 [GenBank: KM249420 - KM249474]; see Additional file 6: Table S2.

### Sequence alignment and phylogenetic analysis

Sequence quality was checked using Finch TV Version 1.4.0 and sequences were edited and aligned using BioEdit [63]. In order to place collected samples into the clade (hereafter species) categorisations of Haine et al. [17], and as a template with which to align newly generated sequences, 23 *P. imperialis* cytb sequences with geographical location data were downloaded from GenBank/EMBI (from accession numbers AJ298439 and AY567594 - AY567638). ITS2 sequences were checked for microsatellites [64] and a small region of 10-16 bp ‘TC’ repeats was identified. Analyses were run with and without this region, which caused no differences in phylogenetic inference, other than a 2% and 3% reduction in support values at two internal nodes. *P. imperialis* ITS2 sequence lengths were between 312-317 bp due to the presence of indels. Indels ranged between 0-4 bp within individual species with no indels found in species 2. Within species 1, a 2 bp indel was diagnostic of specimens from Queensland and New South Wales clades. No obvious pattern was evident among the distribution of indels for other species. Additionally, ITS2 sequence length was 474 bp in the outgroup *Pleistodontes* sp from *F. glandifera* and created indels throughout the alignment. No evidence of pseudogenes or heteroplasmy was noted for mitochondrial regions. Bayesian methods were employed to construct phylogenies using MrBayes [65]. The model of nucleotide substitution for each gene was chosen using jModelTest2 [66]. For mitochondrial markers the TrN + I + G and TPM1uf + G models were chosen for cytb and COI respectively whilst the TPM3uf + G model was chosen for ITS2.

### Species delimitation methods

We used two main strategies, distance (barcoding-type) and phylogeny-based, to delimit species. We applied two distance methods to cytb and COI data: (i) we calculated pairwise genetic distances in PAUP [67] and plotted them in order to determine visually whether a barcode gap was present; (ii) we used jMOTU software [68] to identify the inflection point in the frequency distribution of genetic distances, which is purported to show the barcoding gap. For jMOTU analyses, only sequences without ambiguous base calls were utilized.

As a complementary phylogeny-based analysis on cytb, we identified evolutionary significant units (ESUs) by applying the statistical GMYC method of Pons et al. [69] to the cytb phylogeny, using the R-Splits package [70] in R [71]. The GMYC method requires an ultrametric phylogenetic tree and this was created from cytb data with BEAST software [72]. Models of evolution implemented in BEAST were chosen using Path Sampling and Stepping Stone techniques [73]. These allow the calculation of Bayes factors, which were evaluated using the criteria of Raftery [74], and the HKY + G model was chosen. Two independent runs of 120 million generations were performed, under constant-clock conditions with a constant coalescent model of species evolution. Genealogies and model parameters were sampled once every 6000 iterations. Preliminary runs of the GMYC using only *P. imperialis* sequences yielded non-significant results due to a lack of statistical power, probably due to the limited number of species despite large numbers of individuals. Consequently, we added sequences from four other *Pleistodontes* species and this led to significant results.

Finally, we compared the geographic distributions of the pollinator species across eight regional populations. We used Chi-square tests in R [71] to test null hypotheses that each species occurs at the same frequency in each region.

### Availability of supporting data

Sequence data has been deposited on Genbank whilst population frequencies are in Supporting Information.

## Additional files

Additional file 1: Figure S1 Annotated phylogeny of cytb mtDNA (see Figure 1 legend for details). Additional file 2: Figure S2 Consensus Bayesian topology from COI data for 44 *P. imperialis* individuals. Posterior probabilities are indicated. Tip suffices denote major cytb clade (i.e. species) assigned to each individual by cytb analyses (e.g. C1 = cytb species 1). Additional file 3: Figure S3 Figure 3. Output from jMOTU suggests five *P. imperialis* MOTUs according to percentage sequence discrimination threshold employed for COI sequences. Largest plateau between 18–22 bp indicates barcoding gap. Additional file 4: Figure S4 Modelled TPM1uf + G pairwise distance distribution for 44 *P. imperialis* COI sequences. No barcode gap is evident. Intraspecific distances range between 0–5.6%; interspecific distances between 5.0-13.1%. Additional file 5: Table S1 Population frequency counts for five species of *Pleistodontes imperialis* at eight locations in eastern Australia. Additional file 6: Table S2 Locations, genetic markers sequenced, and GenBank accession numbers for 415 *Pleistondotes imperialis* wasps sequenced in the study.
