# Supplementary figures and images for: Molecular species delimitation of a symbiotic fig-pollinating wasp species complex reveals extreme deviation from reciprocal partner specificity
Source: BMC Evol Biol. 2014 Sep 18;14:189. doi: 10.1186/s12862-014-0189-9 (PMC4172794; doi:10.1186/s12862-014-0189-9)

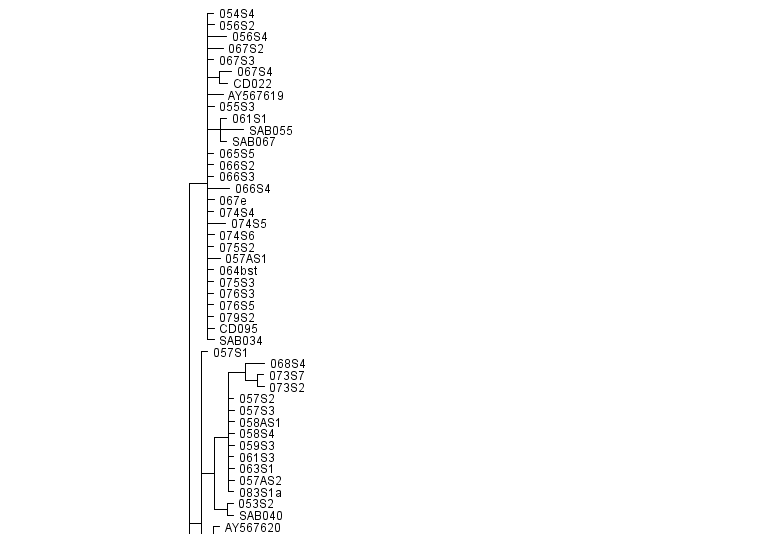

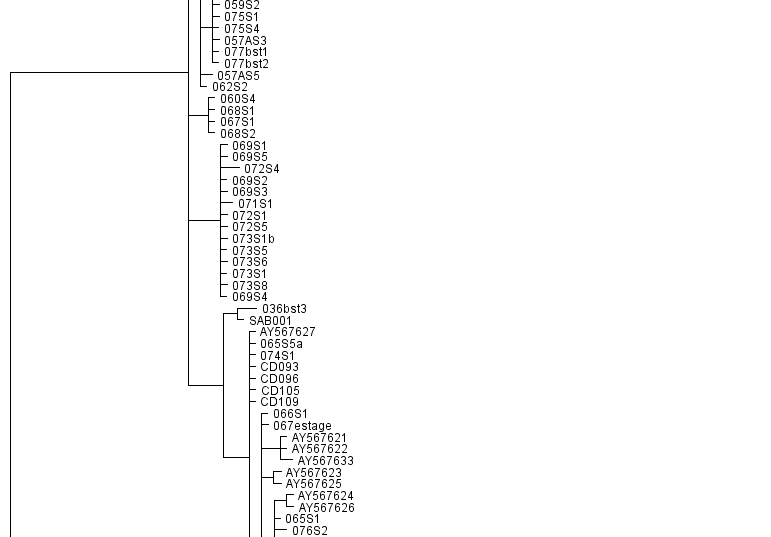


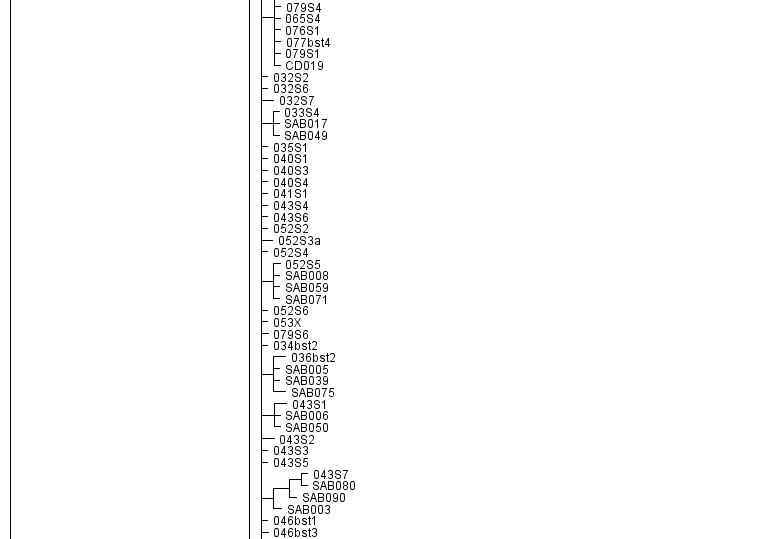

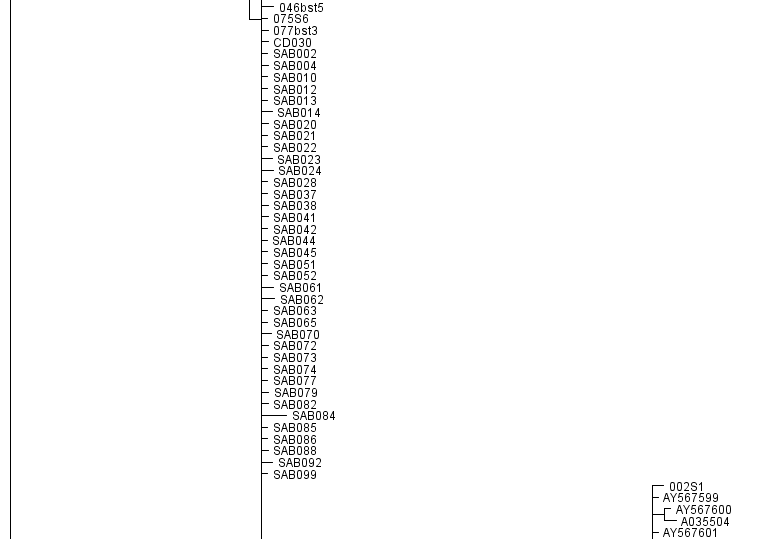


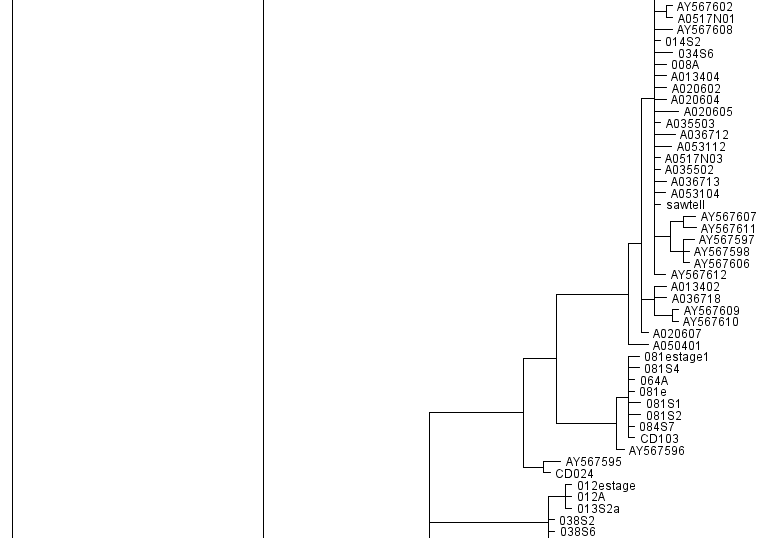

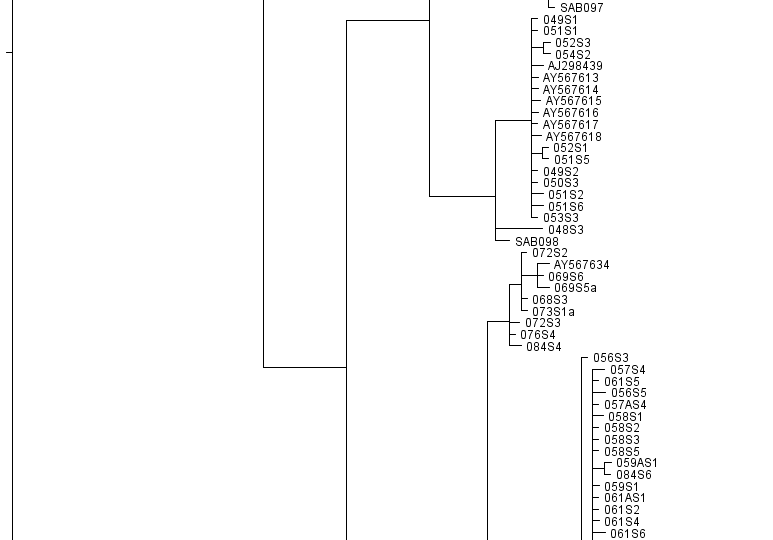


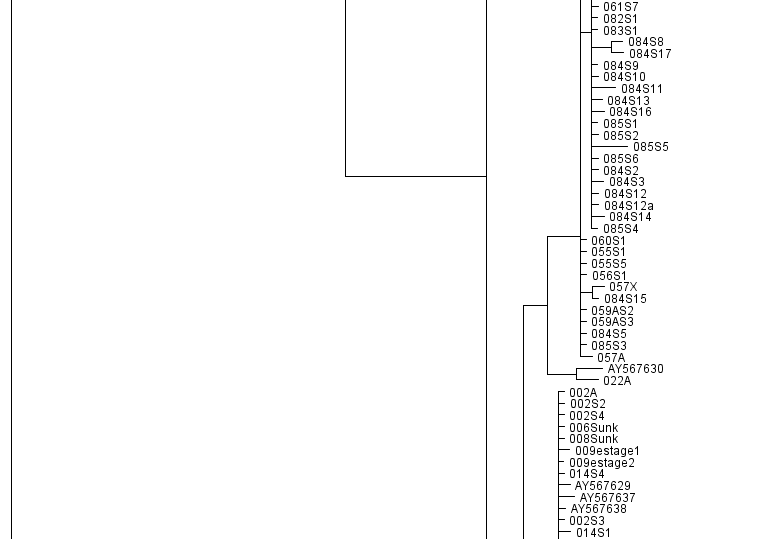

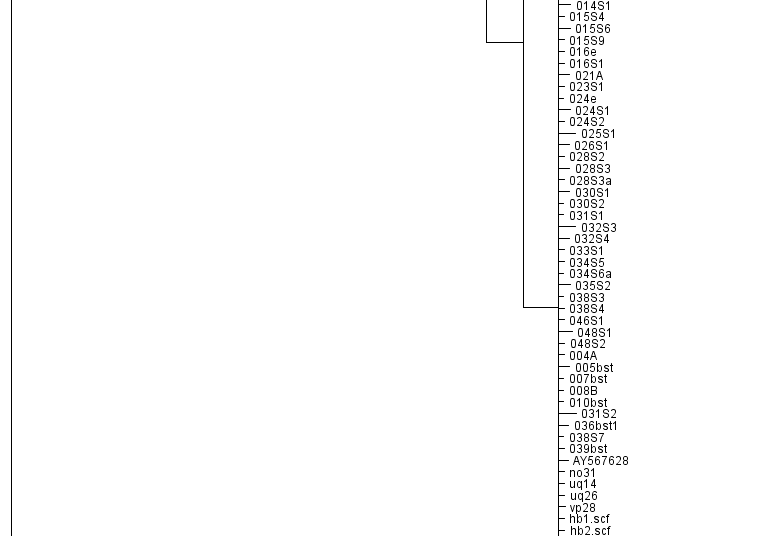


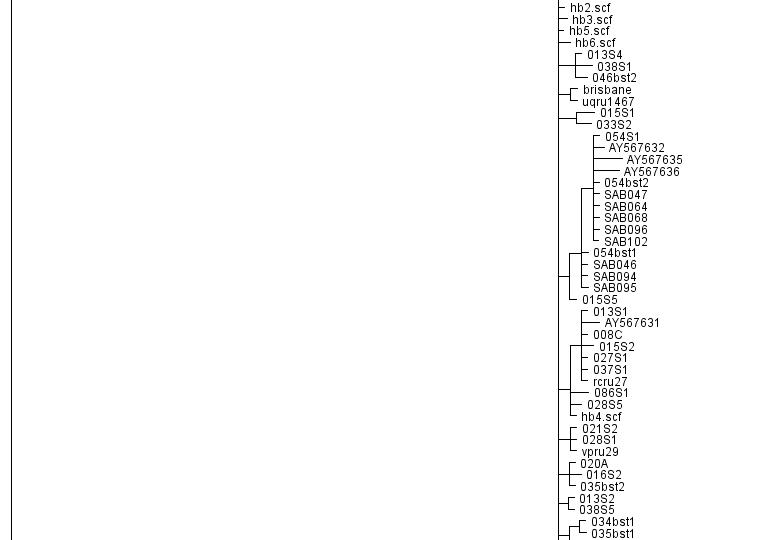

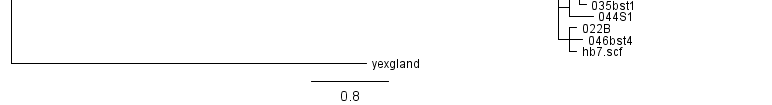

Supplement: Additional file 1: Figure S1 — Annotated phylogeny of cytb mtDNA (see Figure 1 legend for details). [file 12862_2014_189_MOESM1_ESM.docx]

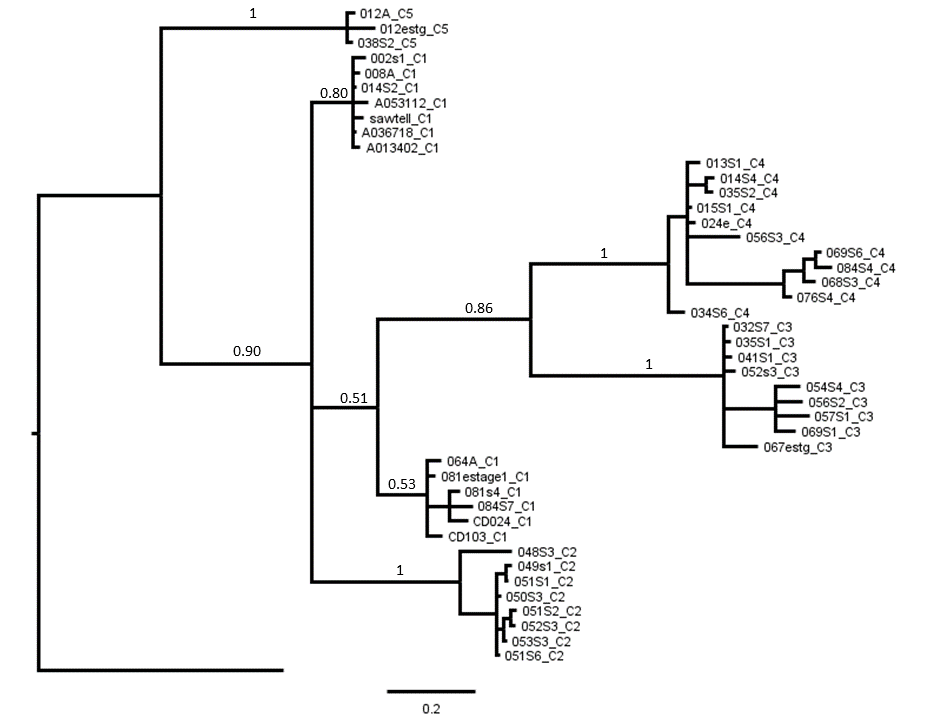

Supplement: Additional file 2: Figure S2 — Consensus Bayesian topology from COI data for 44 P. imperialis individuals. Posterior probabilities are indicated. Tip suffices denote major cytb clade (i.e. species) assigned to each individual by cytb analyses (e.g. C1 = cytb species 1). [file 12862_2014_189_MOESM2_ESM.docx]

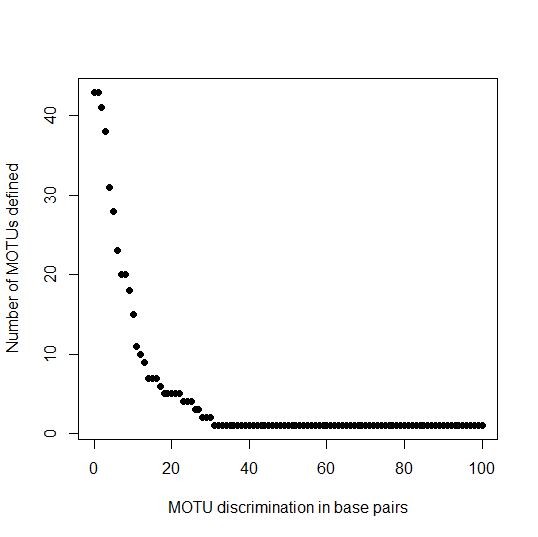

Supplement: Additional file 3: Figure S3 — Figure 3. Output from jMOTU suggests five P. imperialis MOTUs according to percentage sequence discrimination threshold employed for COI sequences. Largest plateau between 18–22 bp indicates barcoding gap. [file 12862_2014_189_MOESM3_ESM.docx]

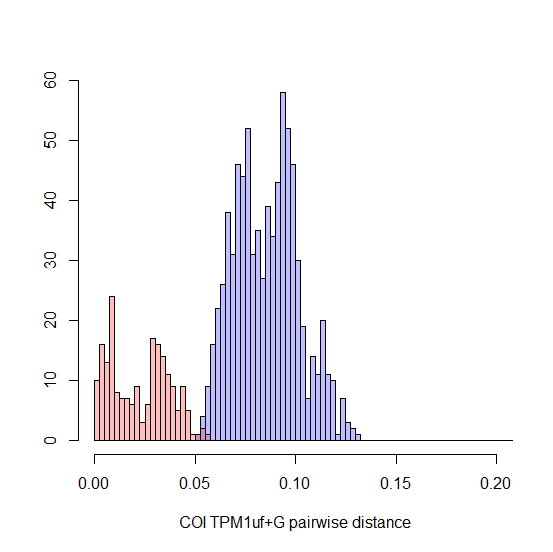

Supplement: Additional file 4: Figure S4 — Modelled TPM1uf + G pairwise distance distribution for 44 P. imperialis COI sequences. No barcode gap is evident. Intraspecific distances range between 0–5.6%; interspecific distances between 5.0-13.1%. [file 12862_2014_189_MOESM4_ESM.docx]
