# Supplementary material for: Molecular species delimitation of a symbiotic fig-pollinating wasp species complex reveals extreme deviation from reciprocal partner specificity
Source: BMC Evol Biol. 2014 Sep 18;14:189. doi: 10.1186/s12862-014-0189-9 (PMC4172794; doi:10.1186/s12862-014-0189-9)
Supplement: Additional file 5: Table S1 — Population frequency counts for five species of Pleistodontes imperialis at eight locations in eastern Australia. [file 12862_2014_189_MOESM5_ESM.docx]

|  | **Population** | | | | | | | |
| --- | --- | --- | --- | --- | --- | --- | --- | --- |
| **Species** | **Atherton Tablelands** | **Chillagoe** | **Townsville** | **Forty Mile Scrub** | **Mackay** | **Capricorn Coast** | **Brisbane** | **New South Wales** |
| **Sp 1** | 11 | 0 | 0 | 0 | 1 | 0 | 7 | 20 |
| **Sp 2** | 0 | 0 | 63 | 0 | 0 | 0 | 0 | 0 |
| **Sp 3** | 51 | 19 | 69 | 20 | 19 | 0 | 3 | 0 |
| **Sp 4** | 2 | 6 | 19 | 48 | 31 | 13 | 43 | 0 |
| **Sp 5** | 0 | 0 | 1 | 0 | 2 | 0 | 3 | 0 |
