# Supplementary material for: Molecular species delimitation of a symbiotic fig-pollinating wasp species complex reveals extreme deviation from reciprocal partner specificity
Source: BMC Evol Biol. 2014 Sep 18;14:189. doi: 10.1186/s12862-014-0189-9 (PMC4172794; doi:10.1186/s12862-014-0189-9)
Supplement: Additional file 6: Table S2 — Locations, genetic markers sequenced, and GenBank accession numbers for 415 Pleistondotes imperialis wasps sequenced in the study. [file 12862_2014_189_MOESM6_ESM.docx]

|  |  | **GenBank Sequence_ID** | | |
| --- | --- | --- | --- | --- |
| **Phylogeny label** | **Location** | **cytb** | **COI** | **ITS2** |
| 002A | Brisbane | KM249475 | - | - |
| 002S1 | Brisbane | KM249476 | KM249375 | KM249420 |
| 002S2 | Brisbane | KM249477 | - | - |
| 002S3 | Brisbane | KM249478 | - | - |
| 002S4 | Brisbane | KM249479 | - | - |
| 004A | Brisbane | KM249480 | - | - |
| 005bst | Noosa | KM249481 | - | - |
| 006Sunk | Noosa | KM249482 | - | KM249421 |
| 007bst | Mudlo | KM249483 | - | - |
| 008A | Kybong | KM249484 | KM249376 | - |
| 008B | Kybong | KM249485 | - | - |
| 008C | Kybong | KM249486 | - | - |
| 008Sunk | Kybong | KM249487 | - | - |
| 009estage1 | Mudlo | KM249488 | - | - |
| 009estage2 | Mudlo | KM249489 | - | - |
| 010bst | Hervey Bay | KM249490 | - | - |
| 012A | Hervey Bay | KM249491 | KM249377 | KM249422 |
| 012estage | Hervey Bay | KM249492 | KM249378 | KM249423 |
| 013S1 | Hervey Bay | KM249493 | KM249379 | KM249424 |
| 013S2 | Hervey Bay | KM249494 | - | KM249425 |
| 013S2a | Hervey Bay | KM249495 | - | KM249426 |
| 013S4 | Hervey Bay | KM249496 | - | - |
| 014S1 | Cooyar Range | KM249497 | - | - |
| 014S2 | Cooyar Range | KM249498 | KM249380 | - |
| 014S4 | Cooyar Range | KM249499 | KM249381 | KM249427 |
| 015S1 | Moore Park | KM249500 | KM249382 | KM249428 |
| 015S2 | Moore Park | KM249501 | - | - |
| 015S4 | Moore Park | KM249502 | - | - |
| 015S5 | Moore Park | KM249503 | - | - |
| 015S6 | Moore Park | KM249504 | - | - |
| 015S9 | Moore Park | KM249505 | - | - |
| 016e | Coconut Point | KM249506 | - | KM249429 |
| 016S1 | Moore Park | KM249507 | - | - |
| 016S2 | Moore Park | KM249508 | - | - |
| 020A | Bluff point | KM249509 | - | - |
| 021A | Bluff point | KM249510 | - | - |
| 021S2 | Bluff point | KM249511 | - | - |
| 022A | Pinnacle Point | KM249512 | - | - |
| 022B | Pinnacle Point | KM249513 | - | - |
| 023S1 | Carmila Beach | KM249514 | - | - |
| 024e | Notch Point | KM249515 | KM249383 | KM249430 |
| 024S1 | Notch Point | KM249516 | - | - |
| 024S2 | Notch Point | KM249517 | - | - |
| 025S1 | Notch Point | KM249518 | - | - |
| 026S1 | Clair View | KM249519 | - | - |
| 027S1 | Sarina Beach | KM249520 | - | - |
| 028S1 | Campwin Beach | KM249521 | - | - |
| 028S2 | Campwin Beach | KM249522 | - | - |
| 028S3 | Campwin Beach | KM249523 | - | - |
| 028S3a | Campwin Beach | KM249524 | - | - |
| 028S5 | Campwin Beach | KM249525 | - | - |
| 030S1 | Coconut Point | KM249526 | - | - |
| 030S2 | Coconut Point | KM249527 | - | - |
| 031S1 | Marlborough | KM249528 | - | - |
| 031S2 | Marlborough | KM249529 | - | - |
| 032S2 | Walkerston | KM249530 | - | - |
| 032S3 | Walkerston | KM249531 | - | - |
| 032S4 | Walkerston | KM249532 | - | - |
| 032S6 | Walkerston | KM249533 | - | - |
| 032S7 | Walkerston | KM249534 | KM249384 | KM249431 |
| 033S1 | Carmila | KM249535 | - | - |
| 033S2 | Carmila | KM249536 | - | - |
| 033S4 | Carmila | KM249537 | - | - |
| 034bst1 | Mirani | KM249538 | - | - |
| 034bst2 | Mirani | KM249539 | - | - |
| 034S5 | Mirani | KM249540 | - | - |
| 034S6 | Mirani | KM249541 | KM249385 | KM249432 |
| 034S6a | Mirani | KM249542 | - | - |
| 035bst1 | Mirani | KM249543 | - | - |
| 035bst2 | Mirani | KM249544 | - | - |
| 035S1 | Mirani | KM249545 | KM249386 | KM249433 |
| 035S2 | Mirani | KM249546 | KM249387 | KM249434 |
| 036bst1 | Mirani | KM249547 | - | - |
| 036bst2 | Mirani | KM249548 | - | - |
| 036bst3 | Mirani | KM249549 | - | - |
| 037S1 | Mackay | KM249550 | - | - |
| 038S1 | Slade Point | KM249551 | - | - |
| 038S2 | Slade Point | KM249552 | KM249388 | KM249435 |
| 038S3 | Slade Point | KM249553 | - | - |
| 038S4 | Slade Point | KM249554 | - | - |
| 038S5 | Slade Point | KM249555 | - | - |
| 038S6 | Slade Point | KM249556 | - | KM249436 |
| 038S7 | Slade Point | KM249557 | - | - |
| 039bst | Eimeo | KM249558 | - | - |
| 040S1 | Seaforth | KM249559 | - | - |
| 040S3 | Seaforth | KM249560 | - | - |
| 040S4 | Seaforth | KM249561 | - | - |
| 041S1 | Finlayson Point | KM249562 | KM249389 | KM249437 |
| 043S1 | St Helens Beach | KM249563 | - | - |
| 043S2 | St Helens Beach | KM249564 | - | - |
| 043S3 | St Helens Beach | KM249565 | - | - |
| 043S4 | St Helens Beach | KM249566 | - | - |
| 043S5 | St Helens Beach | KM249567 | - | - |
| 043S6 | St Helens Beach | KM249568 | - | - |
| 043S7 | St Helens Beach | KM249837 | - | - |
| 044S1 | Wilson Beach | KM249569 | - | - |
| 046bst1 | Bowen | KM249570 | - | - |
| 046bst2 | Bowen | KM249571 | - | - |
| 046bst3 | Bowen | KM249572 | - | - |
| 046bst4 | Bowen | KM249573 | - | - |
| 046bst5 | Bowen | KM249574 | - | - |
| 046S1 | Bowen | KM249575 | - | - |
| 048S1 | Bowen | KM249576 | - | - |
| 048S2 | Bowen | KM249577 | - | - |
| 048S3 | Bowen | KM249578 | KM249390 | KM249438 |
| 049S1 | Bowling Green Bay | KM249579 | KM249391 | - |
| 049S2 | Bowling Green Bay | KM249580 | - | KM249439 |
| 050S3 | Bowling Green Bay | KM249581 | KM249392 | KM249440 |
| 051S1 | Bowling Green Bay | KM249582 | KM249393 | KM249441 |
| 051S2 | Bowling Green Bay | KM249583 | KM249394 | KM249442 |
| 051S5 | Bowling Green Bay | KM249584 | - | - |
| 051S6 | Bowling Green Bay | KM249585 | KM249395 | KM249443 |
| 052S1 | Townsville | KM249586 | - | - |
| 052S2 | Townsville | KM249587 | - | - |
| 052S3 | Townsville | KM249588 | KM249396 | KM249444 |
| 052S3a | Townsville | KM249589 | KM249397 | KM249445 |
| 052S4 | Townsville | KM249590 | - | - |
| 052S5 | Townsville | KM249843 | - | - |
| 052S6 | Townsville | KM249844 | - | - |
| 053S2 | Townsville | KM249591 | - | - |
| 053S3 | Townsville | KM249592 | KM249398 | KM249446 |
| 053X | Townsville | KM249593 | - | - |
| 054bst1 | Toomulla | KM249594 | - | - |
| 054bst2 | Toomulla | KM249595 | - | - |
| 054S1 | Toomulla | KM249596 | - | - |
| 054S2 | Toomulla | KM249597 | - | KM249447 |
| 054S4 | Toomulla | KM249598 | KM249399 | KM249448 |
| 055S1 | Undara | KM249599 | - | - |
| 055S3 | Undara | KM249600 | - | - |
| 055S5 | Undara | KM249601 | - | - |
| 056S1 | Undara | KM249602 | - | - |
| 056S2 | Undara | KM249603 | KM249400 | KM249449 |
| 056S3 | Undara | KM249604 | KM249401 | KM249450 |
| 056S4 | Undara | KM249605 | - | - |
| 056S5 | Undara | KM249606 | - | - |
| 057A | Forty Mile Scrub | KM249607 | - | - |
| 057AS1 | Forty Mile Scrub | KM249608 | - | - |
| 057AS2 | Forty Mile Scrub | KM249609 | - | - |
| 057AS3 | Forty Mile Scrub | KM249610 | - | - |
| 057AS4 | Forty Mile Scrub | KM249611 | - | - |
| 057AS5 | Forty Mile Scrub | KM249612 | - | - |
| 057S1 | Forty Mile Scrub | KM249613 | KM249402 | KM249451 |
| 057S2 | Forty Mile Scrub | KM249614 | - | - |
| 057S3 | Forty Mile Scrub | KM249615 | - | - |
| 057S4 | Forty Mile Scrub | KM249616 | - | KM249452 |
| 057X | Forty Mile Scrub | KM249617 | - | - |
| 058AS1 | Forty Mile Scrub | KM249618 | - | - |
| 058S1 | Forty Mile Scrub | KM249619 | - | - |
| 058S2 | Forty Mile Scrub | KM249620 | - | - |
| 058S3 | Forty Mile Scrub | KM249621 | - | - |
| 058S4 | Forty Mile Scrub | KM249622 | - | - |
| 058S5 | Forty Mile Scrub | KM249623 | - | - |
| 059AS1 | Forty Mile Scrub | KM249624 | - | - |
| 059AS2 | Forty Mile Scrub | KM249625 | - | - |
| 059AS3 | Forty Mile Scrub | KM249626 | - | - |
| 059S1 | Forty Mile Scrub | KM249627 | - | - |
| 059S2 | Forty Mile Scrub | KM249628 | - | - |
| 059S3 | Forty Mile Scrub | KM249629 | - | - |
| 060S1 | Forty Mile Scrub | KM249630 | - | KM249453 |
| 060S4 | Forty Mile Scrub | KM249631 | - | - |
| 061AS1 | Forty Mile Scrub | KM249632 | - | - |
| 061S1 | Forty Mile Scrub | KM249633 | - | - |
| 061S2 | Forty Mile Scrub | KM249634 | - | - |
| 061S3 | Forty Mile Scrub | KM249635 | - | - |
| 061S4 | Forty Mile Scrub | KM249636 | - | - |
| 061S5 | Forty Mile Scrub | KM249637 | - | KM249454 |
| 061S6 | Forty Mile Scrub | KM249638 | - | - |
| 061S7 | Forty Mile Scrub | KM249639 | - | - |
| 062S2 | Forty Mile Scrub | KM249640 | - | - |
| 063S1 | Forty Mile Scrub | KM249641 | - | - |
| 064A | Silver Valley | KM249642 | KM249403 | KM249455 |
| 064bst | Silver Valley | KM249643 | - | - |
| 065S1 | Silver Valley | KM249644 | - | - |
| 065S4 | Silver Valley | KM249645 | - | - |
| 065S5 | Silver Valley | KM249646 | - | - |
| 065S5a | Silver Valley | KM249647 | - | - |
| 066S1 | Silver Valley | KM249648 | - | KM249456 |
| 066S2 | Silver Valley | KM249649 | - | - |
| 066S3 | Silver Valley | KM249650 | - | - |
| 066S4 | Silver Valley | KM249651 | - | - |
| 067e | Irvine Bank-Emuford Road | KM249652 | - | - |
| 067estage | Irvine Bank-Emuford Road | KM249653 | KM249404 | KM249457 |
| 067S1 | Irvine Bank-Emuford Road | KM249654 | - | - |
| 067S2 | Irvine Bank-Emuford Road | KM249655 | - | KM249458 |
| 067S3 | Irvine Bank-Emuford Road | KM249656 | - | - |
| 067S4 | Irvine Bank-Emuford Road | KM249657 | - | - |
| 068S1 | Chillagoe | KM249658 | - | - |
| 068S2 | Chillagoe | KM249659 | - | - |
| 068S3 | Chillagoe | KM249660 | KM249405 | KM249459 |
| 068S4 | Chillagoe | KM249661 | - | - |
| 069S1 | Chillagoe-Mungana | KM249662 | KM249406 | KM249460 |
| 069S2 | Chillagoe-Mungana | KM249663 | - | - |
| 069S3 | Chillagoe-Mungana | KM249664 | - | - |
| 069S4 | Chillagoe-Mungana | KM249665 | - | - |
| 069S5 | Chillagoe-Mungana | KM249666 | - | - |
| 069S5a | Chillagoe-Mungana | KM249667 | - | - |
| 069S6 | Chillagoe-Mungana | KM249668 | KM249407 | KM249461 |
| 071S1 | Mungana | KM249669 | - | - |
| 072S1 | Mungana Truckyards | KM249670 | - | - |
| 072S2 | Mungana Truckyards | KM249671 | - | KM249462 |
| 072S3 | Mungana Truckyards | KM249672 | - | - |
| 072S4 | Mungana Truckyards | KM249673 | - | - |
| 072S5 | Mungana Truckyards | KM249674 | - | - |
| 073S1 | Rookwood | KM249675 | - | - |
| 073S1a | Rookwood | KM249676 | - | - |
| 073S1b | Rookwood | KM249677 | - | - |
| 073S2 | Rookwood | KM249678 | - | - |
| 073S5 | Rookwood | KM249679 | - | - |
| 073S6 | Rookwood | KM249680 | - | - |
| 073S7 | Rookwood | KM249681 | - | - |
| 073S8 | Rookwood | KM249682 | - | - |
| 074S1 | Wolfram Rd nr Bimbulah | KM249683 | - | - |
| 074S4 | Wolfram Rd nr Dimbulah | KM249684 | - | - |
| 074S5 | Wolfram Rd nr Dimbulah | KM249685 | - | - |
| 074S6 | Wolfram Rd nr Dimbulah | KM249686 | - | - |
| 075S1 | Dimbulah Rd nr Mareeba | KM249687 | - | - |
| 075S2 | Dimbulah Rd nr Mareeba | KM249688 | - | - |
| 075S3 | Dimbulah Rd nr Mareeba | KM249689 | - | - |
| 075S4 | Dimbulah Rd nr Mareeba | KM249690 | - | - |
| 075S6 | Dimbulah Rd nr Mareeba | KM249691 | - | - |
| 076S1 | Dimbulah Rd nr Mareeba | KM249692 | - | - |
| 076S2 | Dimbulah Rd nr Mareeba | KM249693 | - | - |
| 076S3 | Dimbulah Rd nr Mareeba | KM249694 | - | - |
| 076S4 | Dimbulah Rd nr Mareeba | KM249695 | KM249408 | KM249463 |
| 076S5 | Dimbulah Rd nr Mareeba | KM249696 | - | - |
| 077bst1 | Dimbulah Rd nr Mareeba | KM249697 | - | - |
| 077bst2 | Dimbulah Rd nr Mareeba | KM249698 | - | - |
| 077bst3 | Dimbulah Rd nr Mareeba | KM249838 | - | - |
| 077bst4 | Dimbulah Rd nr Mareeba | KM249699 | - | - |
| 079S1 | Granite Gorge | KM249700 | - | - |
| 079S2 | Granite Gorge | KM249701 | - | - |
| 079S4 | Granite Gorge | KM249702 | - | - |
| 079S6 | Granite Gorge | KM249703 | - | - |
| 081e | Lake Tinaroo | KM249704 | - | - |
| 081estage1 | Lake Tinaroo | KM249705 | KM249409 | KM249464 |
| 081S1 | Lake Tinaroo | KM249706 | - | - |
| 081S2 | Lake Tinaroo | KM249707 | - | - |
| 081S4 | Lake Tinaroo | KM249708 | KM249410 | KM249465 |
| 082S1 | Forty Mile Scrub | KM249709 | - | - |
| 083S1 | Forty Mile Scrub | KM249710 | - | - |
| 083S1a | Forty Mile Scrub | KM249711 | - | - |
| 084S10 | Forty Mile Scrub | KM249712 | - | - |
| 084S11 | Forty Mile Scrub | KM249713 | - | - |
| 084S12 | Forty Mile Scrub | KM249845 | - | - |
| 084S12a | Forty Mile Scrub | KM249714 | - | - |
| 084S13 | Forty Mile Scrub | KM249715 | - | - |
| 084S14 | Forty Mile Scrub | KM249716 | - | - |
| 084S15 | Forty Mile Scrub | KM249717 | - | - |
| 084S16 | Forty Mile Scrub | KM249718 | - | - |
| 084S17 | Forty Mile Scrub | KM249719 | - | - |
| 084S2 | Forty Mile Scrub | KM249720 | - | - |
| 084S3 | Forty Mile Scrub | KM249846 | - | - |
| 084S4 | Forty Mile Scrub | KM249721 | KM249411 | KM249466 |
| 084S5 | Forty Mile Scrub | KM249722 | - | - |
| 084S6 | Forty Mile Scrub | KM249723 | - | - |
| 084S7 | Forty Mile Scrub | KM249724 | KM249412 | KM249467 |
| 084S8 | Forty Mile Scrub | KM249725 | - | - |
| 084S9 | Forty Mile Scrub | KM249726 | - | - |
| 085S1 | Forty Mile Scrub | KM249727 | - | - |
| 085S2 | Forty Mile Scrub | KM249728 | - | - |
| 085S3 | Forty Mile Scrub | KM249729 | - | - |
| 085S4 | Forty Mile Scrub | KM249730 | - | - |
| 085S5 | Forty Mile Scrub | KM249731 | - | - |
| 085S6 | Forty Mile Scrub | KM249732 | - | - |
| 086S1 | Coconut Point | KM249733 | - | - |
| A013402 | Yarrowyck, NSW | KM249734 | KM249413 | KM249468 |
| A013404 | Yarrowyck, NSW | KM249735 | - | - |
| A020602 | Moonbi, NSW | KM249736 | - | - |
| A020604 | Moonbi, NSW | KM249737 | - | - |
| A020605 | Moonbi, NSW | KM249738 | - | - |
| A020607 | Moonbi, NSW | KM249739 | - | - |
| A035502 | Copeton, NSW | KM249740 | - | - |
| A035503 | Copeton, NSW | KM249741 | - | - |
| A035504 | Copeton, NSW | KM249742 | - | - |
| A036712 | Copeton, NSW | KM249743 | - | - |
| A036713 | Copeton, NSW | KM249744 | - | - |
| A036718 | Copeton, NSW | KM249745 | KM249414 | KM249469 |
| A050401 | Namoi, NSW | KM249746 | - | - |
| A0517N01 | Namoi, NSW | KM249747 | - | - |
| A0517N03 | Namoi, NSW | KM249748 | - | - |
| A053104 | Namoi, NSW | KM249749 | - | - |
| A053112 | Namoi, NSW | KM249750 | KM249415 | KM249470 |
| AJ298439 | Townsville | As given | - | - |
| AY567595 | Atherton | As given | - | - |
| AY567596 | Atherton | As given | - | - |
| AY567597 | Melbourne, Vic | As given | - | - |
| AY567598 | Melbourne, Vic | As given | - | - |
| AY567599 | Brisbane | As given | - | - |
| AY567600 | Brisbane | As given | - | - |
| AY567601 | Brisbane | As given | - | - |
| AY567602 | Brisbane | As given | - | - |
| AY567606 | Adelaide, SA | As given | - | - |
| AY567607 | Sydney, NSW | As given | - | - |
| AY567608 | Sydney, NSW | As given | - | - |
| AY567609 | Melbourne, Vic | As given | - | - |
| AY567610 | Melbourne, Vic | As given | - | - |
| AY567611 | Melbourne, Vic | As given | - | - |
| AY567612 | Melbourne, Vic | As given | - | - |
| AY567613 | Townsville | As given | - | - |
| AY567614 | Townsville | As given | - | - |
| AY567615 | Townsville | As given | - | - |
| AY567616 | Townsville | As given | - | - |
| AY567617 | Townsville | As given | - | - |
| AY567618 | Townsville | As given | - | - |
| AY567619 | Atherton | As given | - | - |
| AY567620 | Atherton | As given | - | - |
| AY567621 | Townsville | As given | - | - |
| AY567622 | Townsville | As given | - | - |
| AY567623 | Atherton | As given | - | - |
| AY567624 | Atherton | As given | - | - |
| AY567625 | Atherton | As given | - | - |
| AY567626 | Atherton | As given | - | - |
| AY567627 | Atherton | As given | - | - |
| AY567628 | Noosa | As given | - | - |
| AY567629 | Brisbane | As given | - | - |
| AY567630 | Noosa | As given | - | - |
| AY567631 | Brisbane | As given | - | - |
| AY567632 | Townsville | As given | - | - |
| AY567633 | Townsville | As given | - | - |
| AY567634 | Atherton | As given | - | - |
| AY567635 | Townsville | As given | - | - |
| AY567636 | Townsville | As given | - | - |
| AY567637 | Brisbane | As given | - | - |
| AY567638 | Brisbane | As given | - | - |
| brisbane | Brisbane | KM249751 | - | - |
| CD019 | Mareeba | KM249752 | - | - |
| CD022 | Mareeba | KM249839 | - | - |
| CD024 | Lake Tinaroo | KM249840 | KM249416 | KM249471 |
| CD030 | Mareeba | KM249847 | - | - |
| CD093 | Yungaburra | KM249753 | - | - |
| CD095 | Yungaburra | KM249754 | - | - |
| CD096 | Yungaburra | KM249755 | - | - |
| CD103 | Yungaburra | KM249848 | KM249417 | KM249472 |
| CD105 | Yungaburra | KM249841 | - | - |
| CD109 | Yungaburra | KM249756 | - | - |
| hb1.scf | Hervey Bay | KM249757 | - | - |
| hb2.scf | Hervey Bay | KM249758 | - | - |
| hb3.scf | Hervey Bay | KM249759 | - | - |
| hb4.scf | Hervey Bay | KM249760 | - | - |
| hb5.scf | Hervey Bay | KM249761 | - | - |
| hb6.scf | Hervey Bay | KM249762 | - | - |
| hb7.scf | Hervey Bay | KM249763 | - | - |
| no31 | Noosa | KM249764 | - | - |
| rcru27 | Redcliffe | KM249765 | - | - |
| SAB001 | Townsville | KM249766 | - | - |
| SAB002 | Townsville | KM249767 | - | - |
| SAB003 | Townsville | KM249768 | - | - |
| SAB004 | Townsville | KM249769 | - | - |
| SAB005 | Townsville | KM249770 | - | - |
| SAB006 | Townsville | KM249771 | - | - |
| SAB008 | Townsville | KM249772 | - | - |
| SAB010 | Townsville | KM249773 | - | - |
| SAB012 | Townsville | KM249774 | - | - |
| SAB013 | Townsville | KM249775 | - | - |
| SAB014 | Townsville | KM249776 | - | - |
| SAB017 | Townsville | KM249777 | - | - |
| SAB020 | Townsville | KM249778 | - | - |
| SAB021 | Townsville | KM249779 | - | - |
| SAB022 | Townsville | KM249780 | - | - |
| SAB023 | Townsville | KM249781 | - | - |
| SAB024 | Townsville | KM249782 | - | - |
| SAB028 | Townsville | KM249783 | - | - |
| SAB034 | Townsville | KM249784 | - | - |
| SAB037 | Townsville | KM249785 | - | - |
| SAB038 | Townsville | KM249786 | - | - |
| SAB039 | Townsville | KM249787 | - | - |
| SAB040 | Townsville | KM249788 | - | - |
| SAB041 | Townsville | KM249789 | - | - |
| SAB042 | Townsville | KM249790 | - | - |
| SAB044 | Townsville | KM249791 | - | - |
| SAB045 | Townsville | KM249792 | - | - |
| SAB046 | Townsville | KM249793 | - | - |
| SAB047 | Townsville | KM249794 | - | - |
| SAB049 | Townsville | KM249795 | - | - |
| SAB050 | Townsville | KM249796 | - | - |
| SAB051 | Townsville | KM249797 | - | - |
| SAB052 | Townsville | KM249798 | - | - |
| SAB055 | Townsville | KM249799 | - | - |
| SAB059 | Townsville | KM249800 | - | - |
| SAB061 | Townsville | KM249801 | - | - |
| SAB062 | Townsville | KM249802 | - | - |
| SAB063 | Townsville | KM249803 | - | - |
| SAB064 | Townsville | KM249804 | - | - |
| SAB065 | Townsville | KM249805 | - | - |
| SAB067 | Townsville | KM249806 | - | - |
| SAB068 | Townsville | KM249807 | - | - |
| SAB070 | Townsville | KM249808 | - | - |
| SAB071 | Townsville | KM249809 | - | - |
| SAB072 | Townsville | KM249810 | - | - |
| SAB073 | Townsville | KM249811 | - | - |
| SAB074 | Townsville | KM249812 | - | - |
| SAB075 | Townsville | KM249813 | - | - |
| SAB077 | Townsville | KM249814 | - | - |
| SAB079 | Townsville | KM249815 | - | - |
| SAB080 | Townsville | KM249816 | - | - |
| SAB082 | Townsville | KM249817 | - | - |
| SAB084 | Townsville | KM249818 | - | - |
| SAB085 | Townsville | KM249819 | - | - |
| SAB086 | Townsville | KM249820 | - | - |
| SAB088 | Townsville | KM249821 | - | - |
| SAB090 | Townsville | KM249822 | - | - |
| SAB092 | Townsville | KM249823 | - | - |
| SAB094 | Townsville | KM249824 | - | - |
| SAB095 | Townsville | KM249842 | - | - |
| SAB096 | Townsville | KM249825 | - | - |
| SAB097 | Townsville | KM249826 | - | - |
| SAB098 | Townsville | KM249827 | - | - |
| SAB099 | Townsville | KM249828 | - | - |
| SAB102 | Townsville | KM249829 | - | - |
| sawtell | Sawtell, NSW | KM249830 | KM249418 | KM249473 |
| uq14 | Brisbane | KM249831 | - | - |
| uq26 | Brisbane | KM249832 | - | - |
| uqru1467 | Brisbane | KM249833 | - | - |
| vp28 | Brisbane | KM249834 | - | - |
| vpru29 | Brisbane | KM249835 | - | - |
| exFglandifera | Sulawesi, Indonesia | KM249836 | KM249419 | KM249474 |
